# Heme Oxygenase-1-Expressing Dendritic Cells Promote Foxp3^+^ Regulatory T Cell Differentiation and Induce Less Severe Airway Inflammation in Murine Models

**DOI:** 10.1371/journal.pone.0168919

**Published:** 2016-12-29

**Authors:** Tzu-Hsuan Wong, Hung-An Chen, Rung-Jiun Gau, Jeng-Hsien Yen, Jau-Ling Suen

**Affiliations:** 1 Graduate Institute of Medicine, College of Medicine, Kaohsiung Medical University, Kaohsiung, Taiwan; 2 Department of Allergy-Immunology-Rheumatology, Chi-Mei Medical Center, Tainan, Taiwan; 3 Biomedical Technology and Device Research Laboratories, Industrial Technology Research Institute, Tainan, Taiwan; 4 Division of Rheumatology, Department of Internal Medicine, Kaohsiung Medical University Hospital, Kaohsiung, Taiwan; 5 Research Center for Environmental Medicine, Kaohsiung Medical University, Kaohsiung, Taiwan; 6 Department of Medical Research, Kaohsiung Medical University Hospital, Kaohsiung, Taiwan; Wayne State University, UNITED STATES

## Abstract

Dendritic cells (DCs) are critical for instructing immune responses toward inflammatory or anti-inflammatory status. Heme oxygenase-1 (HO-1) is known for its cytoprotective effect against oxidative stress and inflammation, suggesting its immune regulatory role in allergic lung inflammation. HO-1 has been implicated in affecting DC maturation; however, its role in DC-mediated T-cell differentiation is unclear. In this study, we demonstrated that HO-1-expressing bone marrow-derived dendritic cells (BM-DCs) displayed tolerogenic phenotypes, including their resistance to lipopolysaccharide (LPS)-induced maturation, high level expression of IL-10, and low T-cell stimulatory activity. In addition, HO-1-expressing DCs were able to induce antigen-specific Foxp3^+^ regulatory T cells (Treg) differentiation in vitro and in vivo. Also, HO-1-expressing DCs modulated the severity of lung inflammatory responses in two murine models of airway inflammation. This study provided evidence supporting the role of HO-1-expressing DCs in tolerance induction and as a potential therapeutic target for allergic asthma as well as other inflammatory diseases.

## Introduction

The lungs are the important organ in contact with external stimuli, including allergens, air pollutants, or infectious agents. Upon stimulation, heme oxygenases-1 (HO-1) activity in the lung represents an important defense mechanism. HO-1 degrades heme into free divalent iron, carbon monoxide and biliverdin, while these metabolites are known for the cytoprotective and anti-inflammatory effects in various disease contexts, including allogenic graft transplantation [1], pregnancy [2], and neutrophilic airway inflammation [3]. Recent studies of HO-1 on lung inflammation and injury clearly show the cytoprotective effect against oxidative stress and lung inflammation by reducing neutrophils infiltration from bone marrow [4, 5]. Understanding the mechanism of HO-1 system in DC’s function may help to design antigen (Ag)-specific therapeutic strategy for lung diseases.

Asthma is a complicated chronic inflammatory disease, which includes Th2-mediated eosinophilic inflammation and Th17-mediated neutrophilic inflammation [6, 7]. Dendritic cell (DC) is the key regulatory cell type for directing Th2 and Th17 differentiation and for the development of allergic diseases [8]. In addition, DCs with tolerogenic property may promote Foxp3^+^ regulatory T cells (Treg) differentiation for immune suppression by secreting TGF-β or expressing inhibitory receptors, such as programmed death ligand 1 (PD-L1) [9–11]. Thus, clarifying the modulatory effect of HO-1 expression in DCs may facilitate the development of Ag-specific tolerance in different types of asthma.

Ag-specific tolerance mediated by Tregs is important for maintaining homeostasis, preventing autoimmunity as well as hypersensitivity. Two subsets of CD4^+^ Tregs have been classified as natural and adaptive Tregs [12, 13]. Natural occurring Tregs develop during normal T-cell maturation in the thymus [14, 15], while adaptive Tregs are developed throughout the course of the immune response in vivo. Although HO-1 induction is associated with elevation of Treg numbers in the context of intestinal inflammation and pregnancy in mice [2, 16]; however, questions remain to be addressed as to whether HO-1 is involved in the development of Tregs through modulating DC differentiation or activity.

Mechanisms that underlie anti-inflammatory effect of HO-1 in the pulmonary inflammation remain largely unknown. Thus, we investigated the mechanisms that contribute to the protective role of HO-1 in murine models of airway lung inflammation. We studied the effect of HO-1 expression on DC differentiation and function and the consequent Ag-specific adaptive Treg differentiation.

## Materials and Methods

### Experimental animals

All animal experiments were performed according to the guidelines of the Institutional Animal Care and Use Committee of the Kaohsiung Medical University. The protocol was approved by the Committee on the Ethics of Animal Experiments of Kaohsiung Medical University (Permit Number: 95162). Female BALB/cByJNarl, and DO11.10 mice, aged 6–8 weeks, were obtained from National Laboratory Animal Center and female C.Cg-Foxp3^tm2Tch^/J (Foxp3^EGFP^) mice from Jackson Lab. All mice were maintained by the Animal Center of Kaohsiung Medical University in a pathogen-free facility. All mice were provided with water and food ad libitum. Animals were anesthetized with intraperitoneal injection of pentobarbital (50–70 mg/kg) before intravenous and intratracheal injections and at sacrifice. The health and condition of all mice in this study were monitored daily prior to sacrifice, and all mice have no clinical signs of ill health. If the mice would have developed signs of severe illness, including weight loss, shortness of breath, and low appetite, they would have been euthanized immediately.

### BM-DCs generation and treatment

Bone marrow cells were cultured with recombinant murine GM-CSF (125 U/ml, Pepro Tech Inc., Rocky Hill, NJ) and 2-mercaptoethanol (50 μM) for 6 days as described previously [17]. Day-6 BM-DCs were further purified with anti-mouse CD11c magnetic beads (Miltenyi Biotec, Sunnyvale, Calif., USA) according to the manufacturer’s instructions. Purified BM-DCs were treated with different concentrations (0–50 μM) of DMSO, Zinc protoporphyrin-IX (ZnPP), Tin protoporphyrin-IX-chloride (SnPP) or cobalt (III) protoporphyrin-IX-chloride (CoPP) for 2 hours and replaced with fresh medium for further 14 hours. LPS (1 μg/ml, *Escherichia coli* O127:B8; Sigma-Aldrich, St. Louis, Mo., USA) was then added as stimuli and cultured for 24 hours. The cells were harvested for phenotypic analysis, western blotting or the analysis of T-cell responses, and supernatants collected for cytokine determination by ELISA (eBioscience, Ireland, UK). The phenotype and purity of BM-DCs were analyzed by flow cytometry (LSR II; BD Biosciences, San Diego, Calif., USA) for the expression of CD11c (G418), MHC class II (M5/114.15.2), CD40 (1C10), CD80 (16-10A1) and CD86 (GL1).

### Western blotting

The treated BM-DCs were lysed in the lyses buffer (3% sodium dodecyl sulfate, 1.67 M urea and 2.7% 2-mercaptoethanol), resolved in a 10% SDS-polyacrylamide gel and electro-transferred onto Hybond-C extra membranes (Amersham, Piscataway, N.J., USA). The membranes were blocked with TBST (50 mM Tris-HCl, 0.15 M NaCl and 0.05% Tween 20) containing 2.5% non-fat milk at room temperature for 1 hour and then incubated with primary antibodies at 4°C overnight. Bound antibodies were detected with peroxidase-labeled secondary antibodies at room temperature for 2 hours, and blots were developed by Western Lightning chemiluminescence reagent (Perkin Elmer, Waltham, Mass., USA). Blots were washed with TBST four times in-between steps. Primary antibodies in these experiments included those for HO-1 and β-actin (Millipore, Billerica, Mass., USA) [18, 19].

### CD4^+^ T cell proliferative response

For mixed lymphocyte reaction, treated BM-DCs from BALB/c mice were cocultured with allogeneic CD4^+^ T cells from C57BL/6 mice at various DC/T ratios for 4 days. For Ag-specific T-cell response, OVA_323-339_-pulsed-BM-DCs from BALB/c mice were cocultured with CD4^+^ T cells from DO11.10 mice for 4 days. During the last 16–18 hours of culture, 1 μCi of [^3^H] thymidine was added to each well. The cells were then harvested and [^3^H] thymidine incorporation was measured in counts per minute (CPM) using a scintillation counter (Topcount, PerkinElmer). The 72-hour supernatant was collected for cytokine determination (ELISA, eBioscience).

### Adaptive Treg differentiation in vitro and in vivo

For in vitro Treg differentiation, treated BM-DCs from BALB/c mice were cocultured with naïve CD4^+^ T cells from syngeneic Foxp3^EGFP^ mice in the presence of anti-CD3 mAb (1 μg/ml, BD Biosciences), rmTGF-β (60 U/ml; Pepro Tech), and rhIL-2 (100 U/ml; Pepro Tech) at ratio 1: 10 for three days. The Foxp3^+^ (EGFP^+^) CD4^+^ T cells were analyzed by flow cytometer (LSR II; BD Biosciences). For in vivo Treg differentiation, naïve CD4^+^ T cells (2 × 10^6^ cells) from DO11.10 (Thy1.2^+^) mice were adoptively transferred into Thy1.1^+^ BALB/c mice on day -1, followed by intravenous or intratracheal administration of OVA_323-339_-pulsed BM-DCs (2 × 10^5^ cells) on day 0. After 5 days, splenocytes or local lymph nodes of lung from the recipient mice were stained with APC-anti-Thy1.2 (53–2.1; eBioscience), FITC-anti-KJ1-26 (KJ1-26; eBioscience), PE-Cy7-anti-CD3 (145-2c11; BD), PerCP-anti-CD4 (RM4-5; eBioscience) and PE-anti-CD25 (PC61; BD) and analyzed by flow cytometer (LSR II). The OVA-specific Tregs (CD3^+^CD4^+^CD25^+^KJ1-26^+^) and non-Ag specific Tregs (CD3^+^CD4^+^CD25^+^KJ1-26^-^) were analyzed with elimination of doubling cells and appropriate isotype controls.

### Establishment and assessment of lung inflammation models

CoPP or DMSO-treated BM-DCs from BALB/c mice were pulsed with OVA (200 μg/ml; grade V; Sigma-Aldrich) and LPS (1 μg/ml) for 24 hours. The harvested BM-DCs were then transferred intratracheally (1 × 10^6^ cells) or intravenously (2 × 10^5^ cells/recipient) into syngeneic naive mice. After 10 days, the recipients were daily challenged with 3% OVA aerosol for 15 minutes for consecutive 4 days. On the day after last challenge, bronchoalveolar lavage fluids (BALFs) and local draining lymph node cells were collected and the cells were stained with PE-Cy7-anti-CD11c (N418; eBioscience), FITC-anti-I-A^d^/I-E^d^ (M5/114.15.2; eBioscience), PE-anti-CCR3 (83101; R&D Systems), APC-anti-CD3 (145-2C11; BD Biosciences) and anti-B220 (RA3-6B2; eBioscience) Abs. The cellular composition of BALF or lymph node cells was determined by flow cytometry (LSR II; BD Biosciences).

### Statistical analysis

Statistical comparisons of data among groups of control and treated BM-DCs were performed with the nonparametric Mann-Whitney U test. Values of p < 0.05 were considered significant. All statistical tests were performed by SPSS for Windows, version 13.0. (SPSS Inc., Chicago, Ill., USA).

## Results

### HO-1 induction inhibits LPS-induced maturation and proinflammatory activity in BM-DCs

HO-1 protein level was initially analyzed when BM-DCs were treated with CoPP in various concentrations as CoPP is widely used as HO-1 inducer in vitro and in vivo. SnPP or ZnPP, two analogs of CoPP, were used as negative controls. We observed that CoPP treatment significantly increased HO-1 protein expression in BM-DCs in a concentration-dependent manner regardless of LPS stimulation (S1 Fig). Instead, SnPP did not have effect on HO-1 expression but had higher toxicity to BM-DCs than CoPP (data not shown).

**Fig 1:**
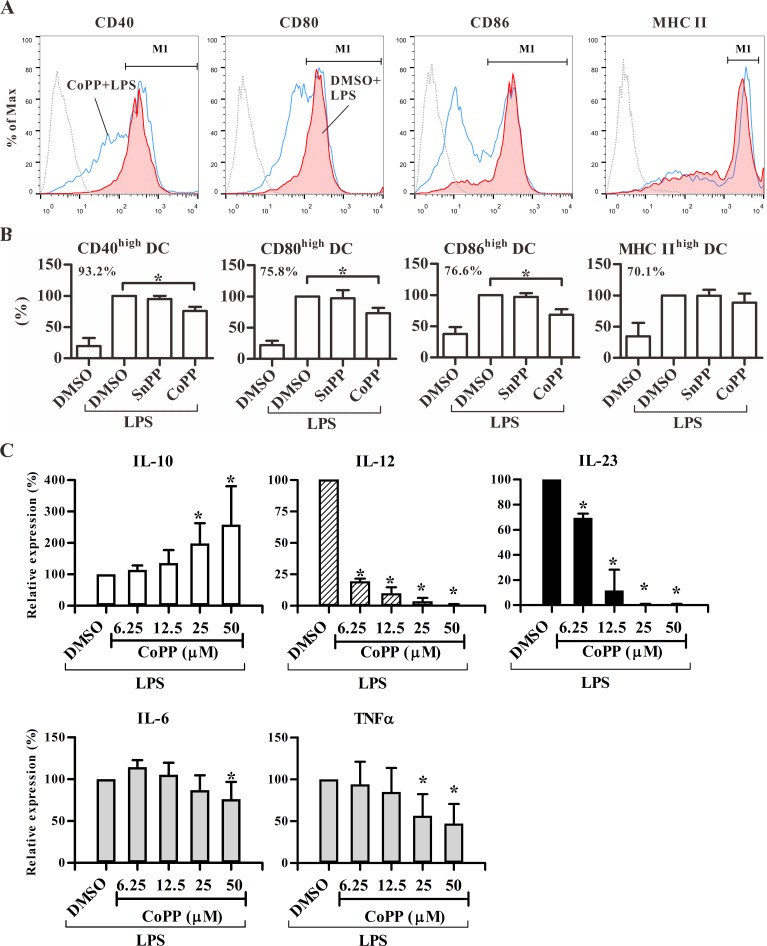
Phenotype and cytokine pattern of CoPP-treated BM-DCs. Treated BM-DCs as indicated from BALB/c mice were stimulated with or without LPS for 24 hours. BM-DCs were harvested for surface marker detection using flow cytometry and supernatants for cytokine analysis using ELISA. (**A**) The histograms shown were gated for live, CD11c^+^ cells. Dotted line: isotype control; red area: DMSO with LPS; blue line: CoPP with LPS. (**B**) Percentages of gated cells (M1 as shown in **A**) from treated BM-DCs were normalized to DMSO with LPS group. The results are shown as mean ± SD of three independent experiments. The number in each graph represents the mean percentage (%) of M1-gated cells in DMSO with LPS group. *p < 0.05 vs. DMSO with LPS. (**C**) The levels of cytokines are shown as relative expression (%) compared to DMSO with LPS (mean ± SD of three to five experiments). * p < 0.05 vs. DMSO with LPS. The cytokine levels of DMSO with LPS (mean ± SD): IL-10: 252 ± 57 pg/ml; IL-12: 1,393 ± 415 pg/ml; IL-23: 97 ± 28 pg/ml. IL-6: 76 ± 5 ng/ml; TNF-α: 1,806 ± 588 pg/ml.

To evaluate the effect of HO-1 expression on DC differentiation, we analyzed the maturation status in BM-DCs. The results showed that induction of HO-1 expression by CoPP significantly inhibited the expression levels of CD40, CD80, CD86 but not MHC class II in BM-DCs in response to LPS (Fig 1A and 1B). In addition, CoPP-conditioned BM-DCs significantly secreted more anti-inflammatory cytokine IL-10 but much less inflammatory cytokines, IL-12, IL-23, IL-6, and TNF-α (Fig 1C) than control cells in a concentration-dependent manner. These data showed that HO-1-expressing DCs displayed an anti-inflammatory phenotype, suggesting their poor stimulatory activity in T-cell responses.

### CoPP-conditioned DCs display tolerogenic phenotype

**Fig 2:**
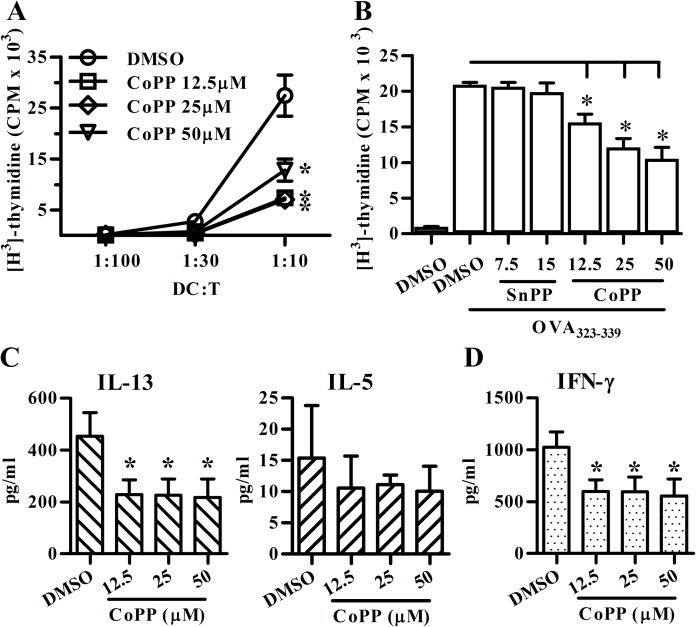
CoPP-conditioned BM-DCs display low CD4^+^ T-cell stimulatory activity and inflammatory cytokine production. (**A**) Treated BM-DCs from BALB/c mice were co-cultured with allogenic CD4^+^ T cells from C57BL/6 mice for 4 days. (**B**) Treated BM-DCs from BALB/c mice were pulsed with OVA_323-339_ and cocultured with syngeneic DO11.10 CD4^+^ T cells for 4 days. Proliferative response was assessed by [^3^H]-thymidine assay. (**C**, **D**) The supernatants from 72-hour DO11.10 CD4^+^ T-DC coculture were analyzed for cytokine levels using ELISA. Data are mean ± SD of three to four independent experiments. * p < 0.05 vs. vehicle (DMSO). All treated BM-DCs in Fig 2 were stimulated with LPS prior to DC-T coculture.

Next, we evaluated the effect of HO-1 expression on DCs in their T-cell stimulatory activity. As shown in Fig 2A, CoPP-treated BM-DCs induced significantly lesser degree of the allogenic T-cell proliferative response compared to the control cells. In addition, OVA_323-339_-pulsed BM-DCs treated with different concentrations of CoPP induced less DO11.10 CD4^+^ T cell proliferation (Fig 2B) as well as IL-13 and IFN-γ secretion (Fig 2C and 2D) as compared to the control. Taken together, these data suggested HO-1-expressing DCs may display tolerogenic phenotypes, including their semi-mature phenotype, poor T-cell stimulatory activity, high anti-inflammatory cytokine IL-10 production as well as low proinflammatory cytokine pattern.

### CoPP-conditioned DCs promote adaptive Treg differentiation

**Fig 3:**
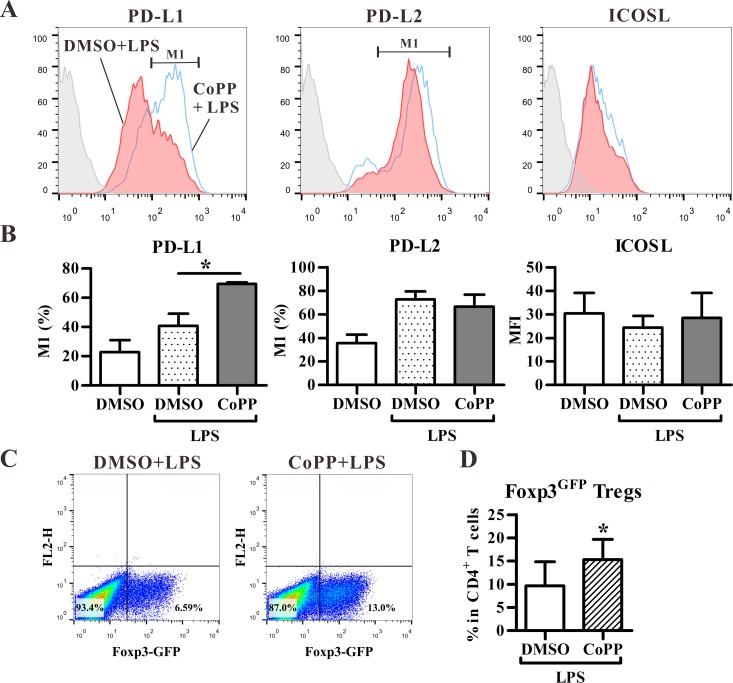
BM-DCs with HO-1 induction enhance PD-L1 expression and adaptive regulatory T cell (Treg) differentiation in vitro. (**A**) BM-DCs treated with DMSO or CoPP were stained with fluorochrome-labeled antibodies for PD-L1, PD-L2, and ICOSL and analyzed by flow cytometry. Gray area: isotype control; red area: DMSO with LPS; blue line: CoPP with LPS. (**B**) The histograms shown are the percentages of PD-L1^+^ or PD-L2^+^ BM-DCs and the mean fluorescence intensity (MFI) of ICOSL^+^ cells as analyzed in **A**. (mean ± SD, n = 3). (**C)** Treated BM-DCs were co-cultured with naïve CD4^+^ T cells from Foxp3^EGFP^ mice for 3 days in the presence of TGF-β and anti-CD3 activating mAb. Representative dot-plots show the expression of Foxp3 (EGFP) on gated CD3^+^CD4^+^ T cells. The treated BM-DCs in **C** were stimulated with LPS. (**D**) The frequency (mean ± SD, n = 5) of Foxp3(EGFP)^+^ Tregs in CD4^+^ T cells as analyzed in **C**. * p < 0.05 vs. DMSO with LPS (Mann-Whitney U test).

Considering that HO-1-expressing DCs may have tolerogenic properties, we further analyzed the expression levels of tolerance-associated molecules, including PD-L1, PD-L2 and inducible costimulator ligand (ICOSL) [20, 21] in CoPP-treated BM-DCs. We observed that CoPP treatment significantly enhanced the expression of PD-L1, but not PD-L2, and ICOSL molecules in BM-DCs (Fig 3A and 3B), suggesting that HO-1-expressing DCs may promote Treg differentiation. Next, Foxp3^EGFP^ mice were utilized to analyze and track Foxp3^+^ T cells at single cell level, as Foxp3^EGFP^ mice co-express Foxp3 and EGFP under the control of endogenous Foxp3 promoter. As shown in Fig 3C and 3D, CoPP-treated BM-DCs significantly promoted the Foxp3^+^ Treg differentiation from naïve CD4^+^ T cells purified from Foxp3^EGFP^ mice in vitro.

**Fig 4:**
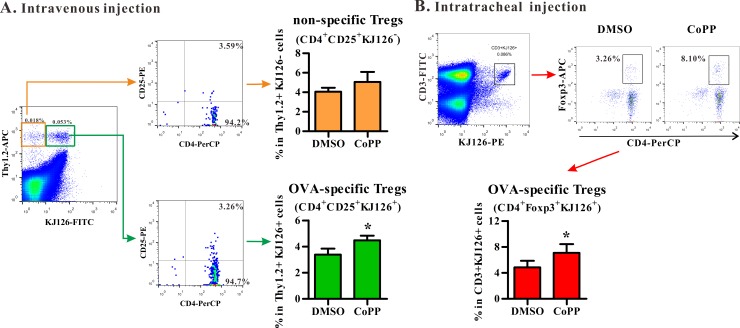
CoPP-conditioned BM-DCs promote Treg differentiation in vivo. (**A**) OVA_323-339_-pulsed BM-DCs and DO11.10 naïve CD4^+^ T cells from Thy1.2^+^ mice were intravenously transferred into Thy1.1^+^ mice for 5 days. Representative dot plots (left) show the expression of CD4 and CD25 on gated Thy1.2^+^KJ1-26^+^ (OVA-specific Tregs, lower panel) or on gated Thy1.2^+^KJ1-26^-^ cells (non-specific Tregs, upper panel). The frequencies (mean ± SD, n = 4) of Tregs are shown (right). (**B**) OVA_323-339_-pulsed BM-DCs and DO11.10 naïve CD4^+^ T cells were intratracheally transferred into BALB/c mice for 5 days. Representative dot plots show the expression of CD4 and Foxp3 on gated CD3^+^KJ1-26^+^ T cells (mean ± SD, n = 5). * p < 0.05 vs. DMSO (Mann-Whitney U test). All treated BM-DCs in Fig 4 were stimulated with LPS.

The in vivo Treg differentiation induced by CoPP-conditioned BM-DCs were further analyzed using two DC delivery routes in the mouse model. The first one is to intravenously deliver DC in vivo. Naïve Thy1.2^+^ CD4^+^ OVA-specific T cells from DO11.10 mice were adoptively transferred into Thy1.1^+^ BALB/c mice. These recipients were then treated intravenously with OVA_323-339_-pulsed BM-DCs with or without HO-1 induction in vitro. The frequencies of Ag-specific Tregs and non-specific Tregs derived from the donor cells in splenocytes from various recipient mice were then compared. Consistent with the results obtained in vitro, CoPP-conditioned BM-DCs promoted the differentiation of OVA-specific (KJ1-26^+^) Tregs, but not the bystander (KJ1-26^-^) Tregs from naïve DO11.10 CD4^+^ T cells (Thy1.2^+^) in vivo (Fig 4A). Furthermore, intratracheal administration was also used to examine the in vivo function of HO-1-expressing DCs. As shown in Fig 4B, the frequencies of OVA-specific Tregs (KJ1-26^+^Foxp3^+^) in local lymph nodes of lungs were significantly higher in mice treated with CoPP-treated BM-DCs than those treated with control cells. These data thus demonstrated that HO-1-expressing BM-DCs were able to promote Ag-specific adaptive Treg differentiation in vivo.

### CoPP treatment suppresses DC-mediated airway inflammation in vivo

**Fig 5:**
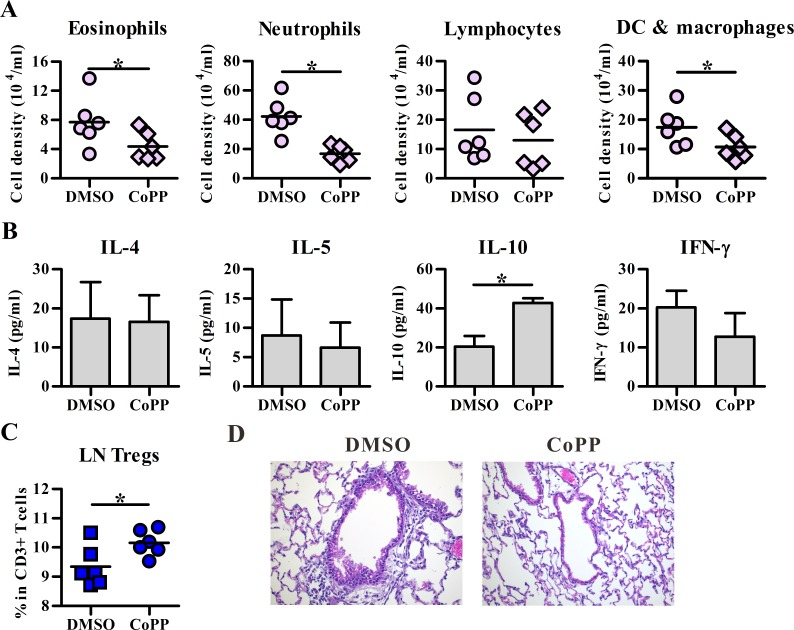
CoPP-conditioned BM-DCs induce less airway inflammation in a neutrophil-dominant model. BALB/c mice were intravenously injected with OVA-pulsed DMSO or CoPP-treated BM-DCs on day 0. Ten days later, both groups of mice were received three daily OVA aerosol challenges. The next day after the last challenge, effector cells in BALFs (**A**), cytokines (**B**) in BALFs and Foxp3^+^ regulatory T cells (Tregs) (**C**) in local lymph nodes (LN) of lung were analyzed by flow cytometry and ELISA, respectively. Data are pooled from one to two independent experiments. The line within the vertical points marks the mean for each group. * p < 0.05 vs. DMSO. DC, dendritic cell. (**D**) Representative section stained with hematoxylin and eosin. The magnification: 200 ×. All treated BM-DCs in Fig 5 were stimulated with LPS.

**Fig 6:**
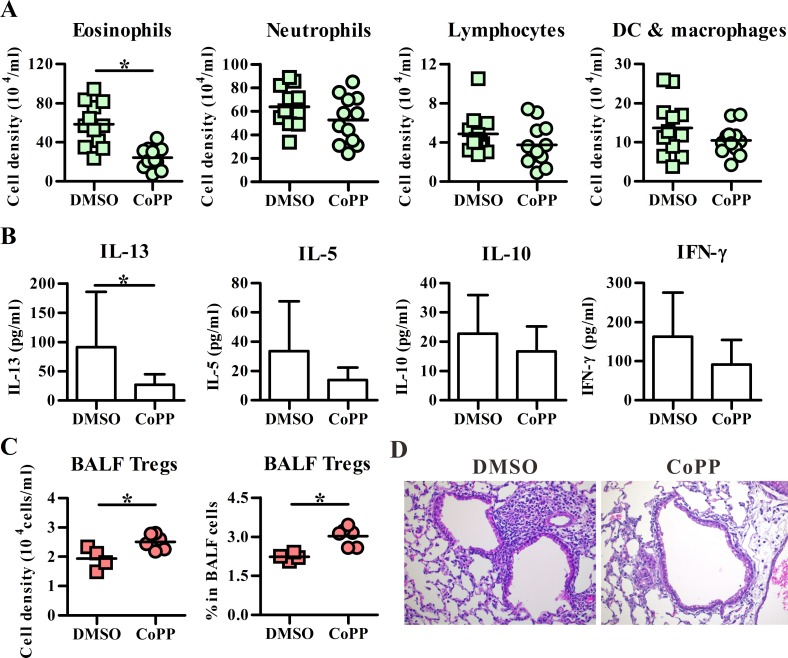
CoPP-conditioned BM-DCs induce less severe eosinophil infiltration in an allergic asthma model. BALB/c mice were intratracheally injected with OVA-pulsed DMSO or CoPP-treated BM-DCs on day 0 and followed by daily OVA aerosol challenges on day 10–12. The next day after the last challenge, effector cells (**A**), cytokines (**B**), and Foxp3^+^ regulatory T cells (Tregs) (**C**) in BALFs were analyzed by flow cytometry and ELISA, respectively. Data are pooled from one to three independent experiments. The line within the vertical points marks the mean for each group. * p < 0.05 vs. DMSO. DC, dendritic cell. (**D**) Representative section stained with hematoxylin and eosin. The magnification: 200 ×. All treated BM-DCs in Fig 6 were stimulated with LPS.

To analyze the in vivo function of HO-1-expressing DCs, airway inflammation was also induced by DCs in two different administration ways. First, transfer of BM-DCs intravenously for inducing neutrophil-dominated lung inflammation was examined, and the results showed that CoPP-treated BM-DCs induced significantly less infiltrating eosinophils and neutrophils, but high BALF IL-10 level as well as high frequency of CD4^+^Foxp3^+^ Tregs in local lymph nodes of lung, as compared to the vehicle controls (Fig 5). In addition, intratracheal injection of BM-DCs also mediated both eosinophil and neutrophil infiltrated lung inflammation (Fig 6A), while CoPP-treated BM-DCs induced a lesser degree of inflammation with low eosinophil infiltration as well as IL-13 expression when compared to vehicle controls (Fig 6B and 6D). Importantly, the number and frequency of CD4^+^Foxp3^+^ Tregs in BALFs were also significantly higher in CoPP group than control group (Fig 6C). Taken together, these data suggested HO-1 expression in DCs may suppress airway inflammation through its ability in promoting adaptive Treg differentiation.

## Discussion

It has been shown that pharmacologic HO-1 induction mediates the anti-inflammatory effects in several models of inflammatory diseases, including asthma [3] and transplantation [22]; however, the interaction of HO-1-expressing DCs and CD4^+^CD25^+^ Tregs remains unclear. This is the first study, to the best of our knowledge, to provide evidence showing that HO-1-expressing DCs are able to induce Ag-specific Foxp3^+^ Treg differentiation and modulate the severity of lung inflammatory responses in vivo. This study provides evidence demonstrating the tolerance-inducing ability of HO-1-expressing DCs, which may offer an experimental basis for the design of therapeutic strategy for allergic asthma as well as other inflammatory diseases.

The detailed molecular mechanisms involved in Treg differentiation induced by HO-1-expressing DCs are largely unknown. This study showed that DCs with HO-1 induction expressed low levels of co-stimulatory molecules (CD40, CD80, CD86), and secreted a high level of IL-10 as well as low levels of IL-12 and IL-23, suggesting the tolerogenic properties of HO-1-expressing DCs. Importantly, these semi-mature DCs with HO-1 induction expressed an increased level of PD-L1. PD-L1 has been reportedly to be critical to the induction of contact-dependent Tregs by vitamin D3-treated human monocyte-derived DCs [23]. The role of HO-1 in regulating PD-L1 expression and in Foxp3^+^ Treg differentiation needs to be further investigated.

Since CoPP can effectively induce tolerogenic DCs, we used Foxp3-EGFP reporter mice to demonstrate the effect of CoPP-treated DCs on adaptive Treg expansion or conversion. It is well known that DCs secrete TGF-β to promote Treg differentiation [24, 25], whereas TGF-β/IL-6 or IL-23 are known to promote proinflammatory Th17 development [26, 27]. In our study, we found that CoPP treated BM-DCs showed similar levels of TGF-β secretion but decreased IL-6 and IL-23 expression, suggesting that CoPP treated DCs may promote adaptive Treg instead of Th17 differentiation.

HO-1 is not only expressed in DCs but also in pulmonary endothelial and epithelial cells as well as alveolar macrophages [28]. Pharmacologic HO-1 induction may mediate anti-inflammatory status through coordinated contribution from several HO-l-expressing cell types; however, among these cell types, only DC has the ability to mediate Ag-specific tolerance in several different mechanisms. First, this study showed that HO-1-expressing DCs can promote Ag-specific adaptive Treg differentiation, at least in part, through PD-L1. Second, IL-10 secreted by HO-1-expressing DCs at high level may enhance type 1 Treg (Tr1) differentiation, as Tr1 has a critical role in controlling peripheral tolerance [29, 30]. Third, HO-1-expressing DCs lost their ability to secrete IL-12 and IL-23, suggesting the inability to mediate Th1 and Th17 responses, respectively. Taken together, this study suggests that induction of HO-1 expression in Ag-bearing DCs can potentially be used to generate Ag-specific tolerance as a cellular therapy for allergic or autoimmune diseases.

In addition to their direct promoting activity on Treg differentiation, HO-1-expressing DCs may act on Ag-specific B cells to affect Treg differentiation in vivo as well. It has been demonstrated that Ag delivery to CD8α^-^ DCs in the marginal zone of mice drives B cells to undergo robust antibody responses to a T cell-dependent Ag [31]. This subset of DCs may uptake Ag by macropinocytosis and transfer it to specific B cells [32]. In addition, B cells can induce T-cell tolerance via different mechanisms, including their ability to secret IL-10 [33]. It suggests, therefore, that HO-1-expressing DCs may promote adaptive Treg differentiation through activating and modulating Ag-specific B cells. The detailed mechanisms through which HO-1-expressing DCs promote adaptive Treg differentiation need to be further elucidated.

Two previous studies as well as our study have observed that HO-1 induction after CoPP treatment of BM-DCs inhibits LPS-mediated maturation [34, 35]. However, there are some conflicting findings among these studies and the present results. Chauveau et al. showed that HO-1 expression also conserves IL-10 expression in rat BM-DCs and human monocyte derived DCs [34]. Consistent with this finding, we demonstrated that CoPP treatment significantly increased IL-10 as well as PD-L1 protein expression of BM-DCs in BALB/c mice. However, in contrast to our results, Mashreghi et al. observed that CoPP treatment suppresses the IL-10 protein as well as PD-L1 mRNA expression in BM-DCs from C57BL/6 mice [35]. The observed discrepancies concerning the expression of tolerance-associated molecules (IL-10 and PD-L1) in these studies might be attributable to species-specific differences. Furthermore, it would be worthy investigating the HO-1 requirement in DC-mediated adaptive Treg differentiation as Mashreghi et al. has demonstrated that inhibition of DC maturation by CoPP does not require HO-1.

In the present study, two models were used for testing the ability of HO-1-expressing DCs on adaptive Treg differentiation; however, different outcomes were noted when two different models were tested. Two possible, but not mutually exclusive, reasons could account for the difference. First, as shown in the present study (Fig 4B) and the previous study [36], intratracheally injected DCs primarily activate and instruct T-cell differentiation in local draining lymph nodes, while DCs delivered intravenously not only accumulate in the lungs and draining lymph nodes, but also disseminate into other organs, such as the spleen ([37], Fig 4A), the latter of which may suggest the possibility of systemic DC’s priming effect on Ag-specific T cell response. Thus, the Ag-induced lung inflammation could be regulated with different kinetics in these two different models of cell transfer. The other possible explanation is the distinct mechanism of action mediated through the use of different models. Ag-specific Tregs induced by intratracheal administration of DCs may predominately act through cell-cell contact dependent mechanism, leading to increased Treg number and frequency noted in the BALFs (Fig 6B and 6C), while the increased frequency of Ag-specific Treg cells induced by intravenous DC transfer may be manifested primarily in local lymph nodes, but not in the BALFs (Fig 5B and 5C). Consequently, the expression of IL-10 is logically expected to show differences in kinetics and tissue locations. Further detailed and comprehensive kinetic analysis is required to properly address these possibilities. Our current study would provide a foundation needed for further investigation.

GM-CSF exhibits pleiotropic activities on multiple cell types and is considered as an inflammatory as well as an anti-inflammatory molecule [38, 39]. Similar to the effect of HO-1, GM-CSF promotes tolerogenic DC differentiation and subsequent adaptive Treg differentiation [40]. However, HO-1 seems not to be involved in the GM-CSF-mediated tolerance, because GM-CSF-differentiated BM-DCs did not express detectable HO-1 protein regardless of LPS stimulation (S1 Fig), but promoted the differentiation of Foxp3^+^ adaptive Treg in the presence of TGF-β (Fig 3D). Whether GM-CSF and HO-1 act, simultaneously or independently, on adaptive Treg differentiation still needs to be further studied.

In summary, we demonstrated, for the first time, that the tolerogenicity of HO-1-expressing DCs may instruct Ag-specific adaptive Treg differentiation, suggesting the potential therapeutic strategy for allergic asthma and other inflammatory diseases.

## Supporting Information

S1 Fig The heme oxygenase-1 (HO-1) induction in bone marrow-derived dendritic cells (BM-DCs). Cobalt (III) protoporphyrin-IX-chloride (CoPP) or tin protoporphyrin-IX (SnPP)-treated BM-DCs from BALB/c mice were stimulated with or without LPS (1 μg/ml) for 24 hours. The HO-1 expression in total cell lysate was analyzed by (**A**) western blotting and (**B**) normalized by β-actin (mean ± SD, *n* = 4). *p < 0.05 vs. vehicle-treated cells.
(TIF)

## Abbreviations

BALF: Bronchoalveolar lavage fluid
BM-DCs: Bone marrow-derived dendritic cells
CoPP: Cobalt (III) protoporphyrin-IX-chloride
DC: Dendritic cell
HO-1: Heme oxygenase-1
ICOSL: Inducible costimulator ligand
PD-L1: Programmed death ligand 1
PD-L2: Programmed death ligand 2
SnPP: Tin protoporphyrin-IX-chloride
Treg: Regulatory T cells
ZnPP: Zinc protoporphyrin-IX

## References

[1] Lee TS, Chau LY. Heme oxygenase-1 mediates the anti-inflammatory effect of interleukin-10 in mice. Nat Med 2002; 8:240–6. 10.1038/nm0302-240 11875494

[2] Schumacher A, Wafula PO, Teles A, El-Mousleh T, Linzke N, Zenclussen ML, et al Blockage of heme oxygenase-1 abrogates the protective effect of regulatory T cells on murine pregnancy and promotes the maturation of dendritic cells. PLoS One 2012; 7:e42301 10.1371/journal.pone.0042301 22900010 PMC3416808

[3] Zhang Y, Zhang L, Wu J, Di C, Xia Z. Heme oxygenase-1 exerts a protective role in ovalbumin-induced neutrophilic airway inflammation by inhibiting Th17 cell-mediated immune response. J Biol Chem 2013; 288:34612–26. 10.1074/jbc.M113.494369 24097973 PMC3843074

[4] Konrad FM, Braun S, Ngamsri KC, Vollmer I, Reutershan J. Heme oxygenase-1 attenuates acute pulmonary inflammation by decreasing the release of segmented neutrophils from the bone marrow. Am J Physiol Lung Cell Mol Physiol 2014; 307:L707–17. 10.1152/ajplung.00145.2014 25172914

[5] Anyanwu AC, Bentley JK, Popova AP, Malas O, Alghanem H, Goldsmith AM, et al Suppression of inflammatory cell trafficking and alveolar simplification by the heme oxygenase-1 product carbon monoxide. Am J Physiol Lung Cell Mol Physiol 2014; 306:L749–63. 10.1152/ajplung.00236.2013 24532288 PMC3989725

[6] Kikuchi S, Nagata M, Kikuchi I, Hagiwara K, Kanazawa M. Association between neutrophilic and eosinophilic inflammation in patients with severe persistent asthma. International archives of allergy and immunology 2005; 137 Suppl 1:7–11. 15947478 10.1159/000085425

[7] Newcomb DC, Peebles RS Jr. Th17-mediated inflammation in asthma. Current opinion in immunology 2013; 25:755–60. 10.1016/j.coi.2013.08.002 24035139 PMC3855890

[8] Willart M, Hammad H. Lung dendritic cell-epithelial cell crosstalk in Th2 responses to allergens. Curr Opin Immunol 2011; 23:772–7. 10.1016/j.coi.2011.09.008 22074731

[9] Steinman RM, Hawiger D, Nussenzweig MC. Tolerogenic dendritic cells. Annu Rev Immunol 2003; 21:685–711. 10.1146/annurev.immunol.21.120601.141040 12615891

[10] Fadilah SA, Cheong SK. Dendritic cell immunobiology and potential roles in immunotherapy. Malays J Pathol 2007; 29:1–18. 19108040

[11] Steinman RM, Banchereau J. Taking dendritic cells into medicine. Nature 2007; 449:419–26. 10.1038/nature06175 17898760

[12] Bluestone JA, Abbas AK. Natural versus adaptive regulatory T cells. Nat Rev Immunol 2003; 3:253–7. 10.1038/nri1032 12658273

[13] Bluestone JA, Tang Q. How do CD4+CD25+ regulatory T cells control autoimmunity? Curr Opin Immunol 2005; 17:638–42. 10.1016/j.coi.2005.09.002 16209918

[14] Sakaguchi S. Naturally arising Foxp3-expressing CD25+CD4+ regulatory T cells in immunological tolerance to self and non-self. Nat Immunol 2005; 6:345–52. 10.1038/ni1178 15785760

[15] Baecher-Allan C, Brown JA, Freeman GJ, Hafler DA. CD4+CD25high regulatory cells in human peripheral blood. J Immunol 2001; 167:1245–53. 11466340 10.4049/jimmunol.167.3.1245

[16] Karimi K, Kandiah N, Chau J, Bienenstock J, Forsythe P. A Lactobacillus rhamnosus strain induces a heme oxygenase dependent increase in Foxp3+ regulatory T cells. PLoS One 2012; 7:e47556 10.1371/journal.pone.0047556 23077634 PMC3471882

[17] Inaba K, Inaba M, Romani N, Aya H, Deguchi M, Ikehara S, et al Generation of large numbers of dendritic cells from mouse bone marrow cultures supplemented with granulocyte/macrophage colony-stimulating factor. J Exp Med 1992; 176:1693–702. 1460426 10.1084/jem.176.6.1693 PMC2119469

[18] Elshabrawy HA, Coughlin MM, Baker SC, Prabhakar BS. Human monoclonal antibodies against highly conserved HR1 and HR2 domains of the SARS-CoV spike protein are more broadly neutralizing. PLoS One 2012; 7:e50366 10.1371/journal.pone.0050366 23185609 PMC3503966

[19] Elshabrawy HA, Fan J, Haddad CS, Ratia K, Broder CC, Caffrey M, et al Identification of a broad-spectrum antiviral small molecule against severe acute respiratory syndrome coronavirus and Ebola, Hendra, and Nipah viruses by using a novel high-throughput screening assay. Journal of virology 2014; 88:4353–65. 10.1128/JVI.03050-13 24501399 PMC3993759

[20] Probst HC, McCoy K, Okazaki T, Honjo T, van den Broek M. Resting dendritic cells induce peripheral CD8+ T cell tolerance through PD-1 and CTLA-4. Nat Immunol 2005; 6:280–6. 10.1038/ni1165 15685176

[21] Ito T, Yang M, Wang YH, Lande R, Gregorio J, Perng OA, et al Plasmacytoid dendritic cells prime IL-10-producing T regulatory cells by inducible costimulator ligand. J Exp Med 2007; 204:105–15. 10.1084/jem.20061660 17200410 PMC2118437

[22] Zhao H, Yoshida A, Xiao W, Ologunde R, O'Dea KP, Takata M, et al Xenon treatment attenuates early renal allograft injury associated with prolonged hypothermic storage in rats. FASEB J 2013; 27:4076–88. 10.1096/fj.13-232173 23759444

[23] Unger WW, Laban S, Kleijwegt FS, van der Slik AR, Roep BO. Induction of Treg by monocyte-derived DC modulated by vitamin D3 or dexamethasone: differential role for PD-L1. European journal of immunology 2009; 39:3147–59. 10.1002/eji.200839103 19688742

[24] Chen W, Jin W, Hardegen N, Lei KJ, Li L, Marinos N, et al Conversion of peripheral CD4+CD25- naive T cells to CD4+CD25+ regulatory T cells by TGF-beta induction of transcription factor Foxp3. J Exp Med 2003; 198:1875–86. 10.1084/jem.20030152 14676299 PMC2194145

[25] Namba K, Kitaichi N, Nishida T, Taylor AW. Induction of regulatory T cells by the immunomodulating cytokines alpha-melanocyte-stimulating hormone and transforming growth factor-beta2. J Leukoc Biol 2002; 72:946–52. 12429716

[26] Chen X, Howard OM, Oppenheim JJ. Pertussis toxin by inducing IL-6 promotes the generation of IL-17-producing CD4 cells. J Immunol 2007; 178:6123–9. 17475838 10.4049/jimmunol.178.10.6123

[27] Bettelli E, Carrier Y, Gao W, Korn T, Strom TB, Oukka M, et al Reciprocal developmental pathways for the generation of pathogenic effector TH17 and regulatory T cells. Nature 2006; 441:235–8. 10.1038/nature04753 16648838

[28] Li N, Venkatesan MI, Miguel A, Kaplan R, Gujuluva C, Alam J, et al Induction of heme oxygenase-1 expression in macrophages by diesel exhaust particle chemicals and quinones via the antioxidant-responsive element. J Immunol 2000; 165:3393–401. 10975858 10.4049/jimmunol.165.6.3393

[29] Levings MK, Gregori S, Tresoldi E, Cazzaniga S, Bonini C, Roncarolo MG. Differentiation of Tr1 cells by immature dendritic cells requires IL-10 but not CD25+CD4+ Tr cells. Blood 2005; 105:1162–9. 10.1182/blood-2004-03-1211 15479730

[30] Levings MK, Sangregorio R, Galbiati F, Squadrone S, de Waal Malefyt R, Roncarolo MG. IFN-alpha and IL-10 induce the differentiation of human type 1 T regulatory cells. J Immunol 2001; 166:5530–9. 11313392 10.4049/jimmunol.166.9.5530

[31] Chappell CP, Draves KE, Giltiay NV, Clark EA. Extrafollicular B cell activation by marginal zone dendritic cells drives T cell-dependent antibody responses. J Exp Med 2012; 209:1825–40. 10.1084/jem.20120774 22966002 PMC3457737

[32] Le Roux D, Le Bon A, Dumas A, Taleb K, Sachse M, Sikora R, et al Antigen stored in dendritic cells after macropinocytosis is released unprocessed from late endosomes to target B cells. Blood 2012; 119:95–105. 10.1182/blood-2011-02-336123 22049514

[33] Ashour HM, Seif TM. The role of B cells in the induction of peripheral T cell tolerance. J Leukoc Biol 2007; 82:1033–9. 10.1189/jlb.0507310 17656652

[34] Chauveau C, Remy S, Royer PJ, Hill M, Tanguy-Royer S, Hubert FX, et al Heme oxygenase-1 expression inhibits dendritic cell maturation and proinflammatory function but conserves IL-10 expression. Blood 2005; 106:1694–702. 10.1182/blood-2005-02-0494 15920011

[35] Mashreghi MF, Klemz R, Knosalla IS, Gerstmayer B, Janssen U, Buelow R, et al Inhibition of dendritic cell maturation and function is independent of heme oxygenase 1 but requires the activation of STAT3. J Immunol 2008; 180:7919–30. 18523255 10.4049/jimmunol.180.12.7919

[36] Lambrecht BN, Pauwels RA, Fazekas De St Groth B. Induction of rapid T cell activation, division, and recirculation by intratracheal injection of dendritic cells in a TCR transgenic model. J Immunol 2000; 164:2937–46. 10706680 10.4049/jimmunol.164.6.2937

[37] Creusot RJ, Yaghoubi SS, Chang P, Chia J, Contag CH, Gambhir SS, et al Lymphoid-tissue-specific homing of bone-marrow-derived dendritic cells. Blood 2009; 113:6638–47. 10.1182/blood-2009-02-204321 19363220 PMC2710920

[38] Bhattacharya P, Thiruppathi M, Elshabrawy HA, Alharshawi K, Kumar P, Prabhakar BS. GM-CSF: An immune modulatory cytokine that can suppress autoimmunity. Cytokine 2015; 75:261–71. 10.1016/j.cyto.2015.05.030 26113402 PMC4553090

[39] Bhattacharya P, Budnick I, Singh M, Thiruppathi M, Alharshawi K, Elshabrawy H, et al Dual Role of GM-CSF as a Pro-Inflammatory and a Regulatory Cytokine: Implications for Immune Therapy. Journal of interferon & cytokine research: the official journal of the International Society for Interferon and Cytokine Research 2015; 35:585–99. 10.1089/jir.2014.0149 PMC4529096 25803788

[40] Bhattacharya P, Gopisetty A, Ganesh BB, Sheng JR, Prabhakar BS. GM-CSF-induced, bone-marrow-derived dendritic cells can expand natural Tregs and induce adaptive Tregs by different mechanisms. J Leukoc Biol 2011; 89:235–49. 10.1189/jlb.0310154 21048215 PMC3024903

