# Supplementary figures and images for: Heme Oxygenase-1-Expressing Dendritic Cells Promote Foxp3+ Regulatory T Cell Differentiation and Induce Less Severe Airway Inflammation in Murine Models
Source: PLoS One. 2016 Dec 29;11(12):e0168919. doi: 10.1371/journal.pone.0168919 (PMC5199094; doi:10.1371/journal.pone.0168919)

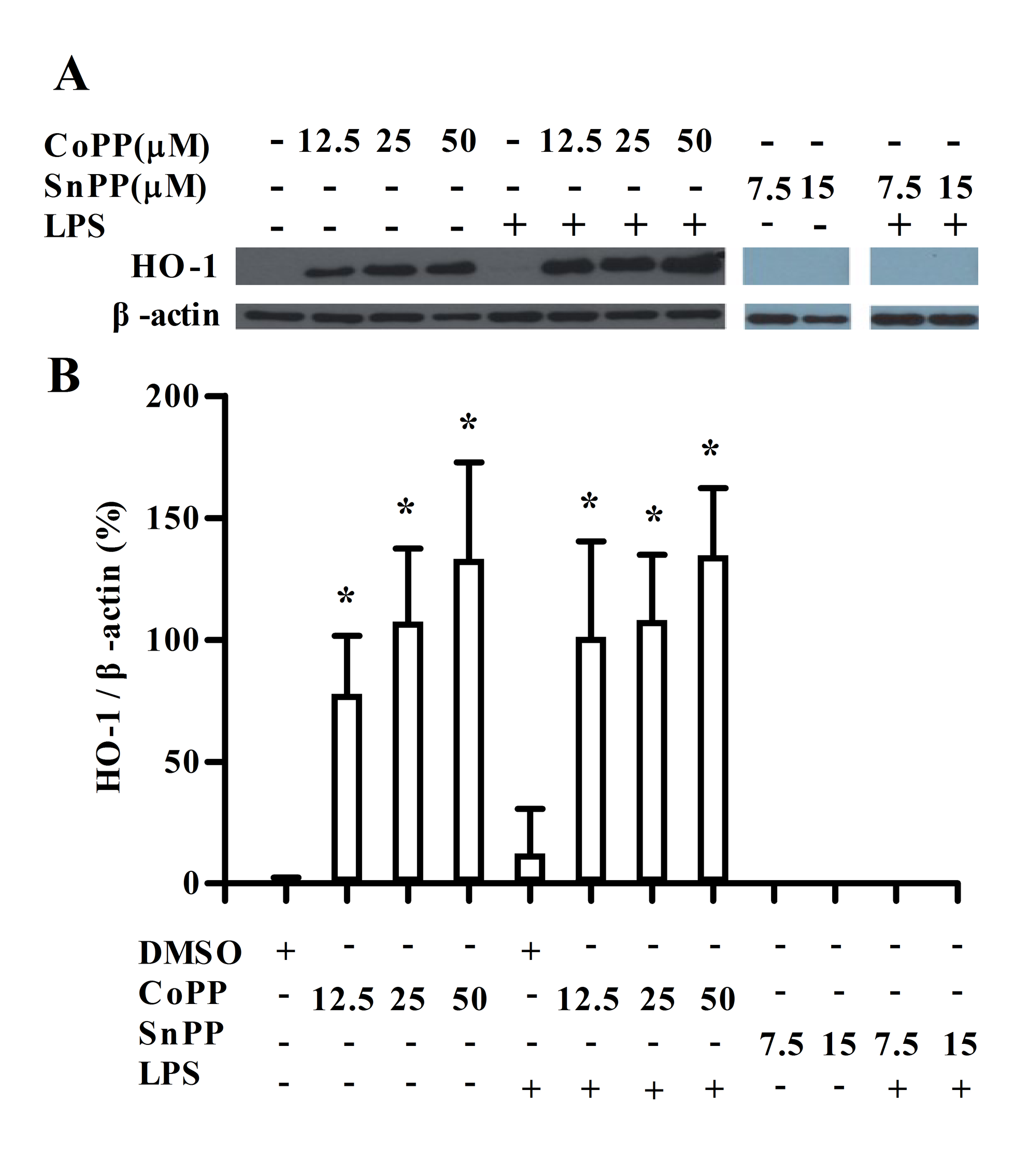

Supplement: S1 Fig — Cobalt (III) protoporphyrin-IX-chloride (CoPP) or tin protoporphyrin-IX (SnPP)-treated BM-DCs from BALB/c mice were stimulated with or without LPS (1 μg/ml) for 24 hours. The HO-1 expression in total cell lysate was analyzed by (A) western blotting and (B) normalized by β-actin (mean ± SD, n = 4). *p < 0.05 vs. vehicle-treated cells. (TIF) [file pone.0168919.s001.tif]
